# Tuning the optoelectronic properties of hematite with rhodium doping for photoelectrochemical water splitting using density functional theory approach

**DOI:** 10.1038/s41598-020-78824-y

**Published:** 2021-01-08

**Authors:** Abdur Rauf, Muhammad Adil, Shabeer Ahmad Mian, Gul Rahman, Ejaz Ahmed, Zia Mohy Ud Din, Wei Qun

**Affiliations:** 1 Department of Physics, University of Peshawar, Peshawar, 25120 Pakistan; 2 Department of Physics, Islamia College University, Peshawar, 25120 Pakistan; 3 Institute of Chemical Sciences, University of Peshawar, Peshawar, 25120 Pakistan; 4 Department of Physics, Abdul Wali Khan University, Mardan, Pakistan; 5 Department of Mechatronic and Biomedical Engineering, Air University Islamabad, Islamabad, Pakistan; 6 Department of Biomedical Engineering, School of Medicine, Keimyung University, Daegu, Republic of Korea

**Keywords:** Physics, Other photonics, Renewable energy, Energy science and technology

## Abstract

Hematite (Fe_2_O_3_) is one of the best candidates for photoelectrochemical water splitting due to its abundance and suitable bandgap. However, its efficiency is mostly impeded due to the intrinsically low conductivity and poor light absorption. In this study, we targeted this intrinsic behavior to investigate the thermodynamic stability, photoconductivity and optical properties of rhodium doped hematite using density functional theory. The calculated formation energy of pristine and rhodium doped hematite was − 4.47 eV and − 5.34 eV respectively, suggesting that the doped material is thermodynamically more stable. The DFT results established that the bandgap of doped hematite narrowed down to the lower edge (1.61 eV) in the visible region which enhanced the optical absorption and photoconductivity of the material. Moreover, doped hematite has the ability to absorb a broad spectrum (250–800) nm. The enhanced optical absorption boosted the photocurrent and incident photon to current efficiency. The calculated results also showed that the incorporation of rhodium in hematite induced a redshift in optical properties.

## Introduction

The immense surge in global population has raised several issues for mankind in recent years. Scientists believe that the energy crisis is one of the most serious issues of our time due to the limited reserves of fossil fuels. Different technological approaches have been suggested to overcome the problem and to meet the energy demands of the world^1^. The photoelectrochemical conversion of water into hydrogen fuel by sunlight with semiconductor electrodes have technological potential because hydrogen is a clean, non-toxic and renewable energy source^2–4^. In photoelectrochemical conversion, the semiconductor electrode plays a very important role, especially in light absorption, charge transport, and charge carrier collection. Different semiconductors like titanium dioxide (TiO_2_), tungsten trioxide (WO_3_)_,_ bismuth vanadate (BiVO_4_), zinc oxide (ZnO) and hematite (Fe_2_O_3_)^5–10^, have been used as photoactive materials for hydrogen production. Among these, hematite is the most promising one due to its narrow bandgap (~ 2**–**2.3) eV^11^, stability in aqueous solution (PH > 3)^12^, photo corrosion^13^ and thermal stability^14^, low-cost synthesis, availability from natural resources, non-toxicity, and environmental friendliness^15^. Hematite also has been applied to develop photocatalysis, gas sensors, lithium-ion batteries, environmental protection, and solar cells^16–22^.

Despite having these attractive features, hematite exhibits low efficiency in photoelectrochemical conversion due to its high resistivity (~ 10^6^ $\Omega$ cm for a single crystal)^23^, high rate of electron–hole recombination^24^, weak electrons and holes mobility (~ 0.01–0.10 cm^2^/V sec)^25^, short-range diffusion length^26^, and small absorption cross-section for incident light^27^. Efforts have been made to improve the photo-response of hematite using suitable dopants to increase its conductivity and absorption coefficient and reduce the electron–holes recombination. The use of nano-sized particles or thin films to increase the diffusion length, mixing of hematite with other oxides like titanium dioxide, and the use of catalysts are some other strategies to improve the surface kinetics^28,29^. Doping of hematite has long been considered a means to improve the conductivity and water splitting activity of hematite. However, the exact role of dopant is still a topic of interest for researchers.

Computational studies have been carried out to gain insights into the role of doping in hematite to enhance its optoelectronic properties. Density Functional Theory (DFT) calculations were conducted by Joseph et al. to analyze copper doped^30^ and {01–12} hematite layers for efficient water splitting and to measure its thermodynamic stability based on energy from the surface and energy from its formation. They reported that copper doping decreases the bandgap of hematite and puts its valence and conduction band in a favorable position suggesting water splitting without external biasing voltage^31^. Doping of elements from group-IV (Ge, Si, & Sn) in hematite suggests that due to the saturated coordination in the matrix substitutional doping results in better optoelectronic properties as compared with interstitial doping making doped hematite a good candidate as a photo-anode material for water oxidation^32^. Richard et al. used the GGA + U approach and investigated the structural, electronic, magnetic, and optical properties of aluminum-doped hematite and found that the incorporation of aluminum changes the band structure of hematite and also influences the magnetic dipole moment near the defective site^33^. Haijun Pan et al. have investigated 4d transition metals doped hematite for its performance in the photoelectrochemical process using DFT, and the predicted properties were verified experimentally. They reported that transition metals doped hematite results in high optical absorption and electrical conductivity in the visible part of the solar spectrum which will enhance the PEC activity^34^. Daniel et al. have studied water oxidation on a bare hematite (0001) surface as well as hematite (0001) surface covered with a monolayer of gallium trioxide (Ga_2_O_3_) using density functional theory and described intermediate reactions involving lattice oxygen bound to water adsorbed oxygen, both on the bare and covered surfaces and found many layer terminations in surface energy) very similar^35^. They reported over potential very close for all these terminations, 0.8 V on the bare surface and 0.95 V on the gallium trioxide surface. They concluded that the conduction band relating to the gallium trioxide layer is significantly higher in energy than that of the hematite conduction band consequently, the photogenerated electrons on the surface are not accessible for recombination.

In this study, we have used rhodium substitution doping in hematite, rhodium oxide (Rh_2_O_3_) has a bandgap of (1.2–1.4) eV with a corundum structure similar to hematite. The similar structure of rhodium oxide to that of hematite reveals that it can reduce the bandgap of pure hematite significantly and will enhance the absorption of light in the visible and near-infrared regions of light. Furthermore, it is expected that the use of rhodium as a dopant will not produce any other crystalline phases in the host hematite crystal^36,37^.

To investigate the effects of rhodium doping in hematite, DFT simulations were carried out in this work to calculate the photoconductivity, optical absorption, bandgap energy, dielectric function and reflectivity for both pure and doped hematite.

## Computational methods and setup

In this work pure and rhodium doped hematite have been studied using DFT through the SIESTA simulations package^38^. The generalized gradient approximation (GGA) with the exchange correlation authors, Revised Perdew-Burke Ernzehof (RPBE)^39^ has been used for the optimization of all geometries. A hybrid DFT with 75% local density approximation (LDA) and 25% generalized gradient approximation (GGA) has been widely used in the past to calculate the structural parameters of various materials. However, transition metals have highly correlated electrons and most of the DFT techniques incorrectly compute the electronic properties of the metal oxides. For such materials, the above-mentioned techniques also incorrectly describe the columbic repulsion in the localized d state electrons^40^. In order to accurately calculate the electronic band structure and density of states (DOS) we have implemented the DFT + U technique in this work. We have used a $1\times 1\times 1$ supercell with a cutoff energy of 200Ry,$3\times 3\times 1$ k-points for geometry optimization and 6 k$\times 6\times 6$-points for the electronic properties calculations. The pseudo-atomic orbitals (PAO’s)^41^ basis set with double zeta potential (DZP) have been applied to all atoms in this configuration**.**

## Results and discussion

### Geometry analysis of pure & rhodium doped hematite

**Figure 1:**
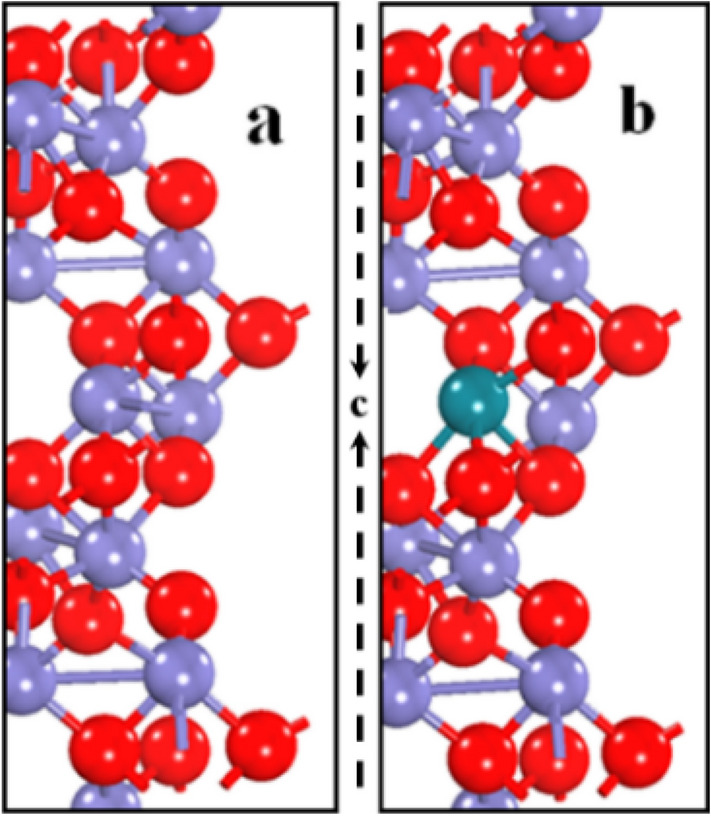
Unit cell geometry of pure (**a**) and rhodium Doped (**b**) hematite. The ball colors, blue, red and green represent Fe, O and Rh atoms respectively, with ‘c’ along the vertical axis.

**Figure 2:**
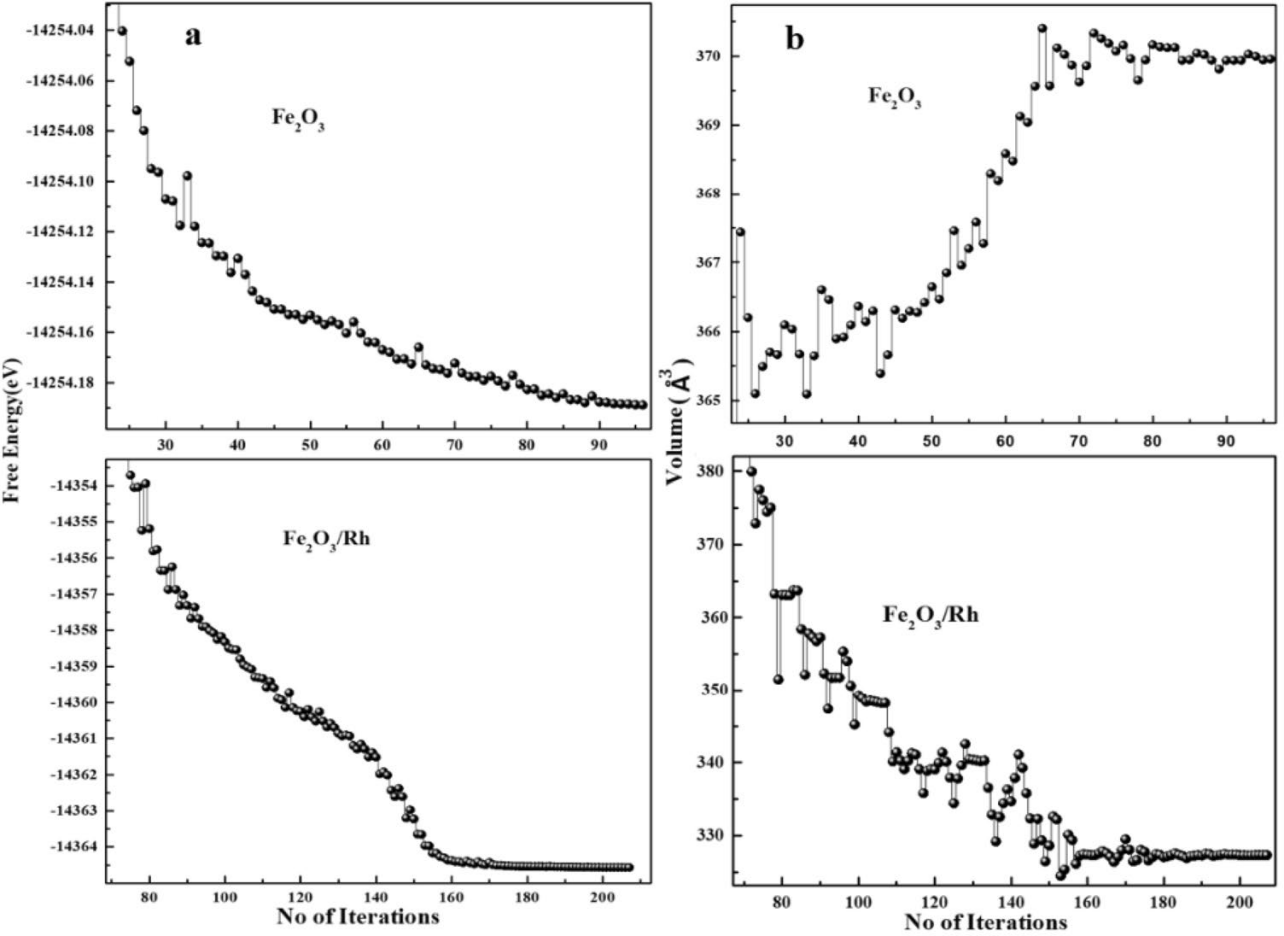
Free energy (**a**) and volume (**b**) iterations no. variation in pure and doped hematite.

This study is based on substitutional doping in the hexagonal structure of pure hematite. The host iron atom was replaced with a rhodium atom, as shown in Fig. 1. The relaxed geometry of doped hematite showed stability of the structure (via a decrease in total energy) compared to that of pure hematite indicating the favorable incorporation of rhodium. It has been observed that all systems tend to a phase of minimal free energy to a maximally stable state^42^. In this work, the free energy for pure and doped systems has been calculated to investigate the thermodynamic stability of the electrode material for photoelectrochemical (PEC) activity. The trends in the free energy for pure and doped hematite with the number of iterations are depicted in Fig. 2a. where it can be observed that the free energy of rhodium doped hematite at the optimized stage has the largest negative value of free energy indicating its stability.

The calculated lattice parameters of pure hematite, average Fe–Fe distance and Fe-Rh distance are given in Table 1. The inter-atomic distance is significantly affected due to the insertion of rhodium atom. An increase in inter-atomic distance is due to the higher atomic radius of Rh^43^. The insertion of an impurity atom in the host material causes an expansion in the volume of the doped hematite. The variation in the volume of pure and rhodium doped hematite versus the number of iterations during geometry optimization through the SIESTA simulation package is shown in Fig. 2b. The thermodynamic stability was also evaluated by calculating the formation energy of pristine and rhodium doped hematite using the following relation^30^.

$${\text{E}}_{{\text{f}}} \left( {{\text{Fe}}_{{2}} {\text{O}}_{{3}} /{\text{Rh}}} \right) = {\text{E}}_{{{\text{tot}}}} \left( {{\text{Fe}}_{{2}} {\text{O}}_{{3}} } \right) - {\text{2E}}_{{{\text{Fe}}}} - {\text{3E}}_{{\text{O}}} - {\text{E}}_{{{\text{Rh}}}} .$$

E_f_(Fe_2_O_3_/Rh) is the formation energy of Fe_2_O_3_/Rh, E_tot_(Fe_2_O_3_) is the total energy of hematite, E_Fe_ is the energy of iron atom, E_O_ represents the energy of oxygen atom and E_Rh_ is the energy of rhodium atom. The formation energies of pristine and rhodium doped hematite were respectively − 4.47 eV and − 5.34 eV representing that rhodium doped hematite synthesis is favorable in the laboratory.

**Table1:** Comparison of lattice constant a (Å), c (Å) and average Fe–Fe (Å) and Fe–Rh (Å) distance with other DFT and experimental results.

Pure & doped hematite calculations					
Calculations	GGA	Hybrid DFT	DFT + U	Ref. DFT	Exp
a(Å)	4.848	4.705	5.04	5.008^a^, 5.071^a^	5.035^c^
c(Å)	13.805	10.85	12.203	13.87^a^, 13.903^a^	13.747^c^
Fe–Fe (Å)	2.77	2.855	3.04	2.86^b^, 2.85^b^, 2.89^b^	2.896^d^
Fe–Rh (Å)	3.130	3.249	4.58	–	–

^a^Ref^32^.

^b^Ref^33^.

^c^Ref^44^.

^d^Ref^45^.

### Electronic structure

**Figure 3:**
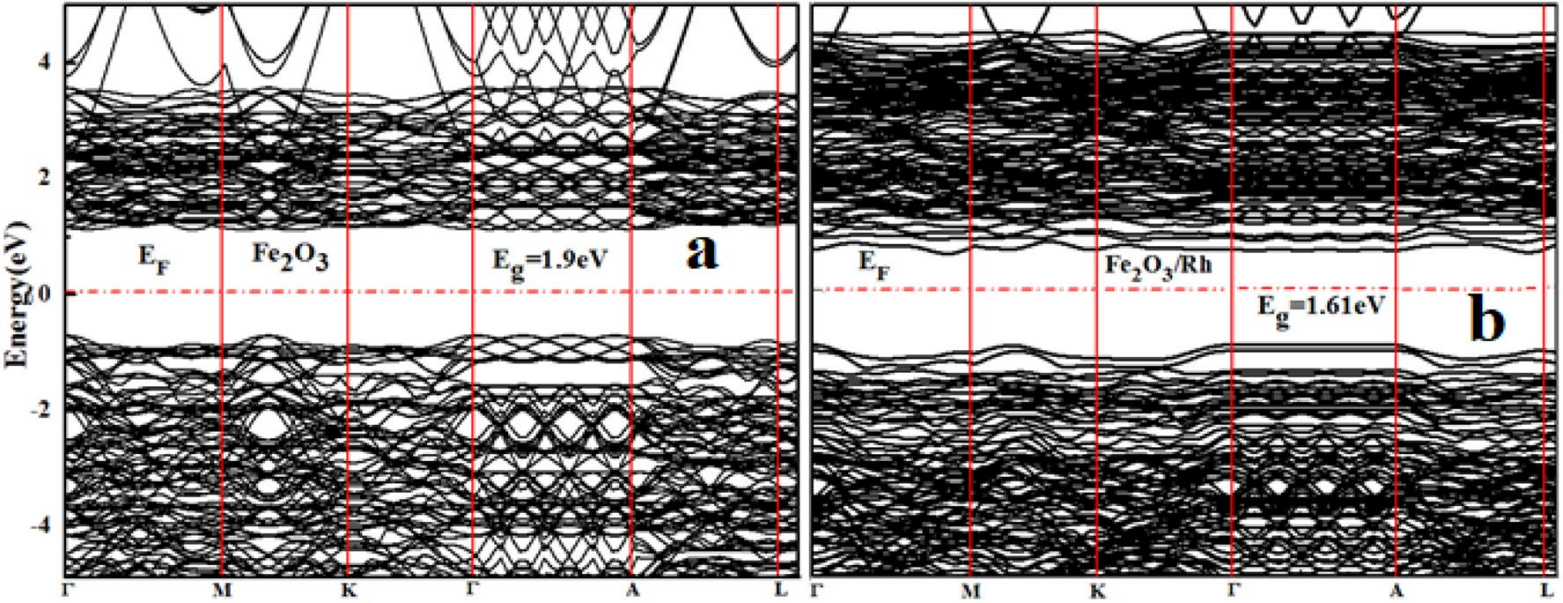
DFT + U method Band structures of (**a**) pure and (**b**) Rh doped hematite.

The electronic properties of pure and doped hematite have been calculated using GGA, DFT + U and hybrid DFT. The results obtained from both GGA and hybrid DFT underestimated the bandgap while the DFT + U provide accurate results consistent with experimental and other DFT calculations. The electronic structures of pure and doped hematite are shown in Fig. 3a,b respectively.

**Figure 4:**
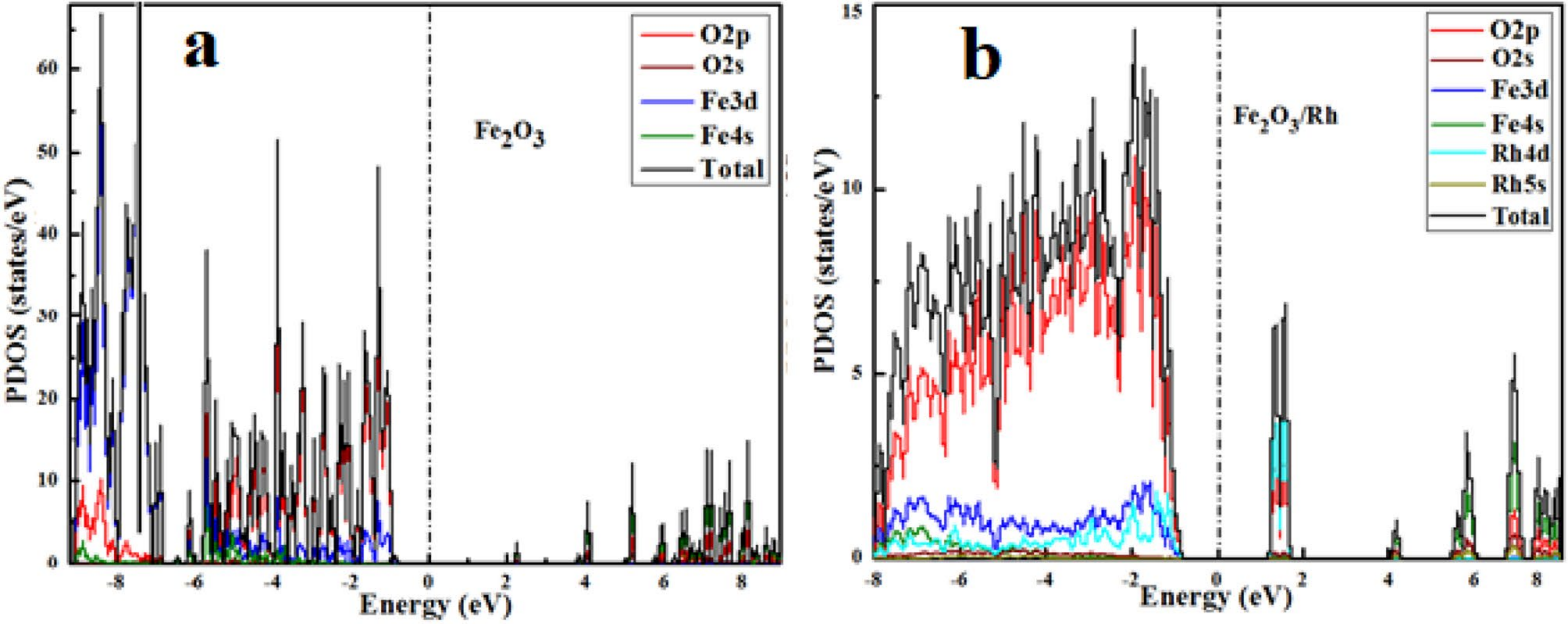
DFT + U method projected density of states for pure (**a**) and Rh doped hematite (**b**).

The projected density of states (PDOS) of pure and rhodium doped hematite (Fig. 4a,b), respectively indicate the influence of rhodium on the electronic properties of hematite. Figure 4a suggests that maximum contribution at the upper valence band near the Fermi level is attributed to the occupied O-2p states while a small contribution was observed from the occupied Fe-3d and O-2s. A lower conduction band showed a maximum contribution from Fe-4s states. Similarly, the valence band of rhodium doped hematite contained the occupied O-2p state along with the mixed states of both Fe-3d and Rh-4d, as shown in Fig. 4b. At the conduction band edge near the fermi level Rh-4d and O-2p energy levels dominated while a smaller contribution in the upper conduction region from the Fe-3d was observed. The highly occupied states near the Fermi level have high impact on the optical properties of the material and can cause a transition of electrons to some vacant energy states in the conduction band upon absorption of incident photon energy.

**Figure 5:**
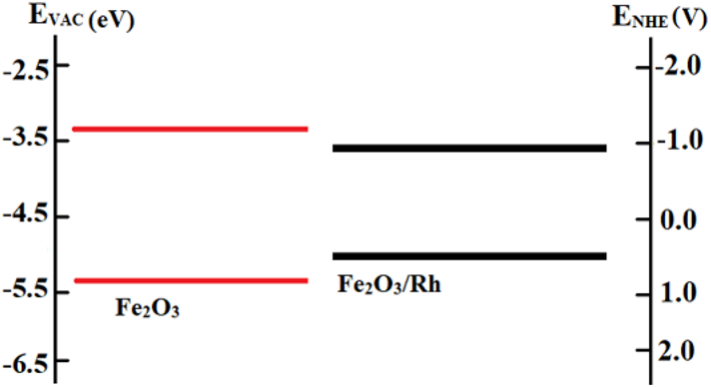
The band edge positions of pure and Rh doped hematite at vacuum level E_VAC_ relative to normal hydrogen electrode potential E_NHE_.

In the photocatalytic process, the position of the valence band maximum (VBM) and the minimum conduction band (CBM) is very important for the redox reaction. Figure 5 shows the energy of the valence band and conduction band of pristine and rhodium doped hematite. The lower and upper red and the black horizontal color lines respectively represent the position of the VBM and CBM for pristine and rhodium doped hematite. Shifts in the valence band and the conduction band can be seen from Fig. 5 representing the creation of optimal energy states and the modification of VBM and CBM due to the incorporation of rhodium.

### Real and imaginary parts of the dielectric function

**Figure 6:**
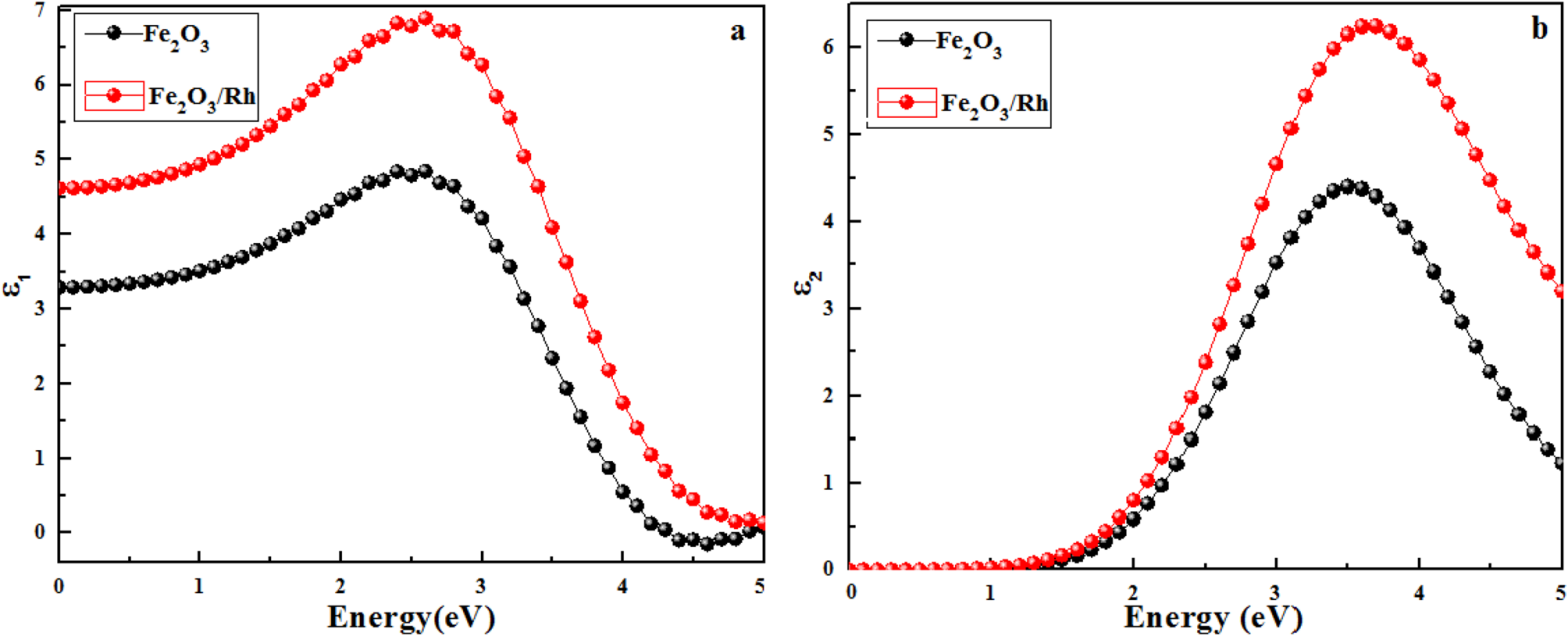
Incident photon energy variations in (**a**) real and (**b**) imaginary part of the dielectric function for both pure and doped hematite.

The electronic properties of materials in response to the electric field of incident radiation depend upon the dielectric function, $\in \left( {\upomega } \right) = \in_{ 1 } \left( {\upomega } \right) + {\text{i}} \in_{2} \left( {\upomega } \right)$ where the real part of the dielectric function (${\in }_{1}(\upomega ))$ is related to the electronic polarizability of the material and imaginary part of dielectric function (${\in }_{ 2 }(\upomega ))\mathrm{ is related to}$ the electronic absorption in the material due to the irradiation of light. The values of ${\in }_{1}(\upomega )$ and ${\in }_{2}(\upomega )$ were calculated using the SIESTA package for pure and rhodium doped hematite and their variation with a change in the energy levels of incident light is shown in Fig. 6.

${\in }_{1}$ has a peak value of 4.84 at 2.6 eV for pure hematite while rhodium incorporation in hematite enhanced the peak value to 6.892 at the same energy level implying that rhodium doping in hematite results in enhanced electronic polarization in the material as compared to pure hematite. The polarizability of the material has potential applications in energy storage devices^46^. The maximum values of ${\in }_{2}$ are 4.402 and 6.115 at 3.5 eV for pure hematite and rhodium doped hematite respectively (Fig. 5b) representing higher energy loss in the material. It can also be concluded that the 3.5 eV peak results due to the transition of an electron from O-2p occupied states in the upper valence band near the Fermi level to the vacant states of Fe-3d at the lower end of the conduction band while the peak corresponding to rhodium doped hematite is observed at 3.7 eV. However, this peak is attributed to the transition of electrons from the inner occupied states to some unoccupied states in the conduction band. Overall, doping improved the real and imaginary parts of the dielectric function in the entire range of the solar energy spectrum including substantial enhancement in the visible range. The values of the real and imaginary parts of the dielectric function in the visible region are shown in Table 2.

**Table 2:** Real (${\in }_{1}(\upomega ))$ and Imaginary ${(\in }_{2}(\upomega ))$ parts of the dielectric function in the visible region at E _photon_ = 1.65 eV ($\lambda$ $\approx$ 750 nm).

Specimen	ε_1_	Experiment	ε_2_	Experiment
Fe_2_O_3_	4.111	6.865^47^	0.405	0.3197^47^
Fe_2_O_3_/Rh	4.852	–	1.088	–

### Optical absorption and photoconductivity

Absorption of a broad solar spectrum by using the photo-electrode material is one of the necessary conditions for solar energy is driven water splitting. Beer’s law explains the decrease in the intensity of light passing through a medium:

1 $${\text{I}}\left( {\text{z}} \right) = {\text{I}}_{0} {\text{exp }}\left( { - \alpha {\text{ z}}} \right)$$

where α represents the quantity of incident light absorbed in a unit length of the medium^48^. I_0_ and I (z) are the intensities at the surface and the thickness of the medium, respectively.

**Figure 7:**
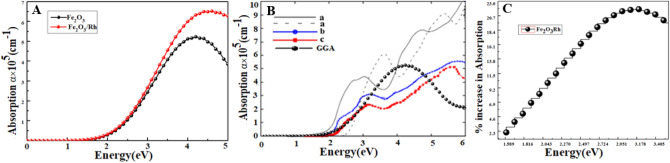
(**A**) Absorption coefficient was calculated using GGA method within DFT for pure and rhodium doped hematite, (**B**) optical absorption of hematite compared with other DFT (a) and experimental results (b,c). The DFT results have been taken from Simone et al.^49^ and experimental data from the work of Gardner et al.^50^ & Marusak et al.^51^ (**C**) a percentage increase in absorption of rhodium doped hematite in the visible range .

Figure 7A shows that rhodium doping in hematite resulted in enhanced absorption from infrared to visible and ultraviolet regions of the electromagnetic spectrum. As discussed earlier, rhodium doping significantly narrowed the bandgap of hematite which in turn enhanced the absorption coefficient by absorbing photons of longer wavelengths. Enhancement of optical absorption in the visible range of solar energy can increase the solar energy conversion efficiency by 32% due to the sensitization of photoelectrode material in the visible range, and the calculations performed in this work agree with the reference data^52^.

The calculated optical absorption compared with other DFT and experimental results are shown in Fig. 7B. The percentage increase in optical absorption in the visible range of rhodium doped hematite compared with pure hematite is shown in Fig. 7C^28^. The dominant prevalence in optical absorption has several benefits such as increase in photogenerated electrons and photoconversion efficiency^53^. The optical absorption of hematite with dopant in the UV–Vis region has been improved and is consistent with experimental data^54^. The calculated optical absorption compared with other DFT and experimental results are shown in Fig. 7B. The percentage increase of optical absorption in the visible range of rhodium doped hematite compared with pure hematite is shown in Fig. 7C.

**Figure 8:**
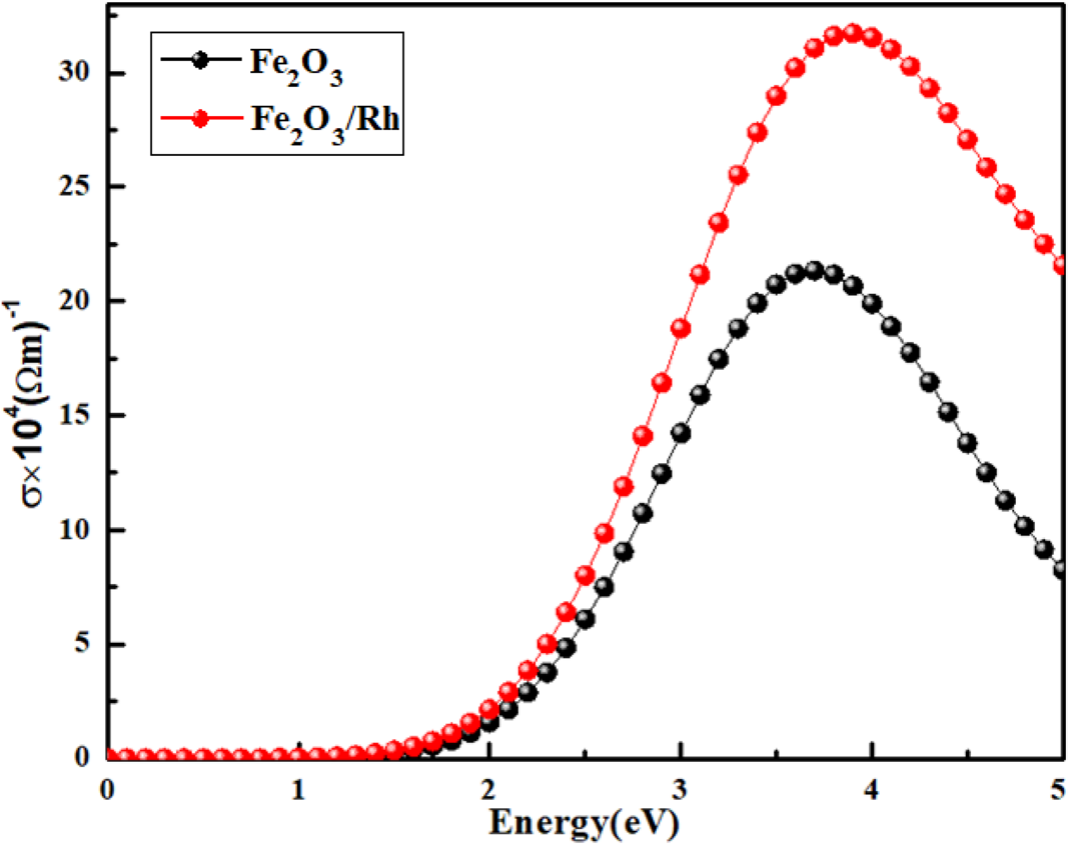
Calculated optical conductivity of pure and rhodium doped hematite using GGA approximation in DFT.

The increase in conductivity of hematite in the visible range has technological importance in photoelectrochemical water splitting and optoelectronic devices. Figure 8 shows that the incorporation of rhodium in hematite increased conductivity due to the transition of electrons from the occupied O-2p state in the upper valence band to the empty states of Fe-3d & Rh-4d in the lower edge of the conduction band. In other words, the charge transport efficiency improved substantially, subsequently improving the photoelectrochemical activity of material^55^. The values of the absorption coefficient and conductivity at different energies are listed in Table 3.

**Table 3:** Absorption coefficient and conductivity in the visible range.

Specimen	α × 10^5^ (cm^−1^)	E _Photon_ (eV)	σ × 10^5^ (Ωm)^−1^
Fe_2_O_3_	2.226	1.8	0.811
Fe_2_O_3_	2.127	3.2	0.693
Fe_2_O_3_/Rh	2.339	1.8	0.992
Fe_2_O_3_/Rh	2.589	3.2	0.892

**Figure 9:**
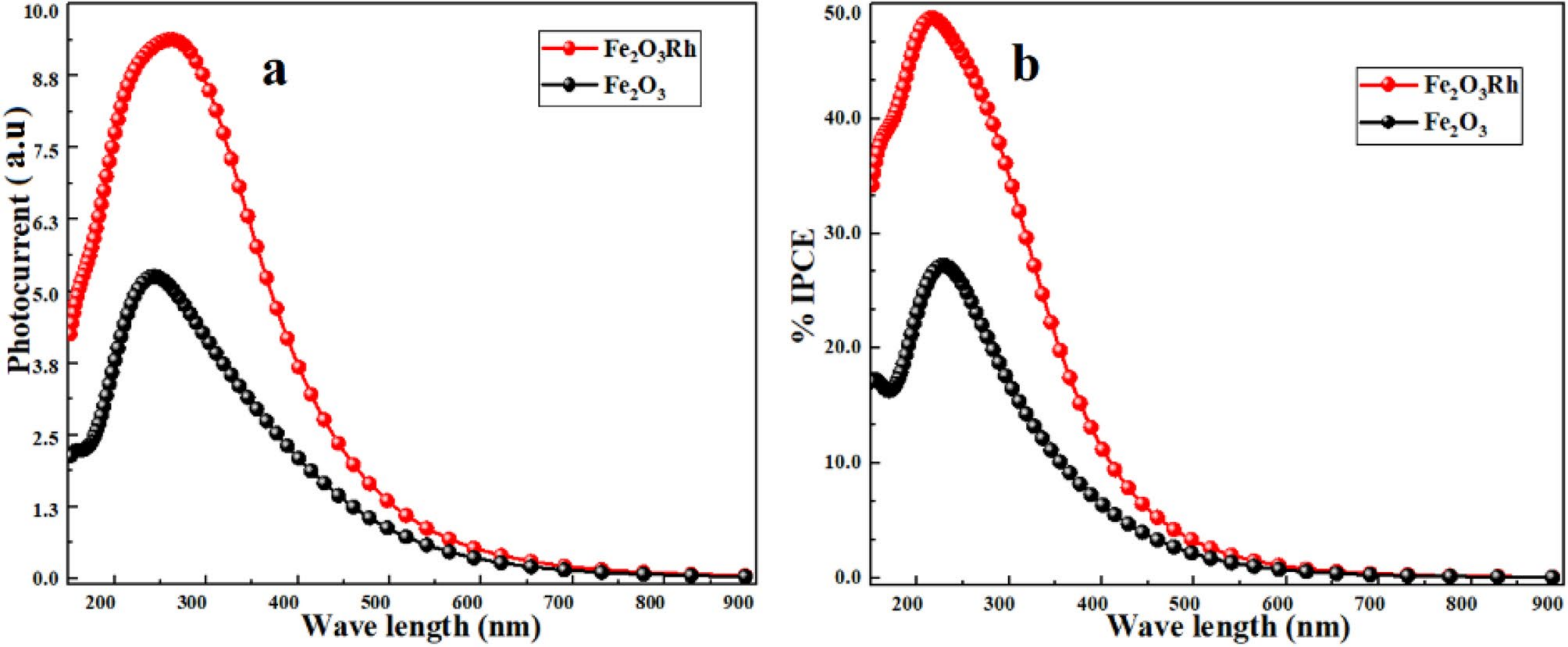
The enhancement in (**a**) photocurrent and (**b**) IPCE for rhodium doped hematite.

The enhancement in optical absorption and optical conductivity has improved photogenerated current which improves the incident photon to current efficiency (IPCE) as shown in Fig. 9a,b.

Photocurrent and incident photon to current efficiency (IPCE) have been measured for pure and rhodium doped hematite are shown in Fig. 9. Figures 9a,b demonstrate that photocurrent and IPCE have been enhanced with doping in (250–800) nm in accordance with our absorption spectra for rhodium doped hematite and compatible with experiments^28,56^. In optically active materials, absorption and scattering of light significantly depend on the refractive index and extinction coefficient. The complex refractive index $\stackrel{\sim }{n}$ is given by:

2 $$\ddot{\tilde{n}} = n + i\kappa$$

where n is the ordinary refractive index and $\kappa$ is the absorption index or extinction coefficient^57^. The behavior of the refractive index and extinction coefficient before and after rhodium doping in hematite is shown in Fig. 8a,b, respectively.

**Figure 10:**
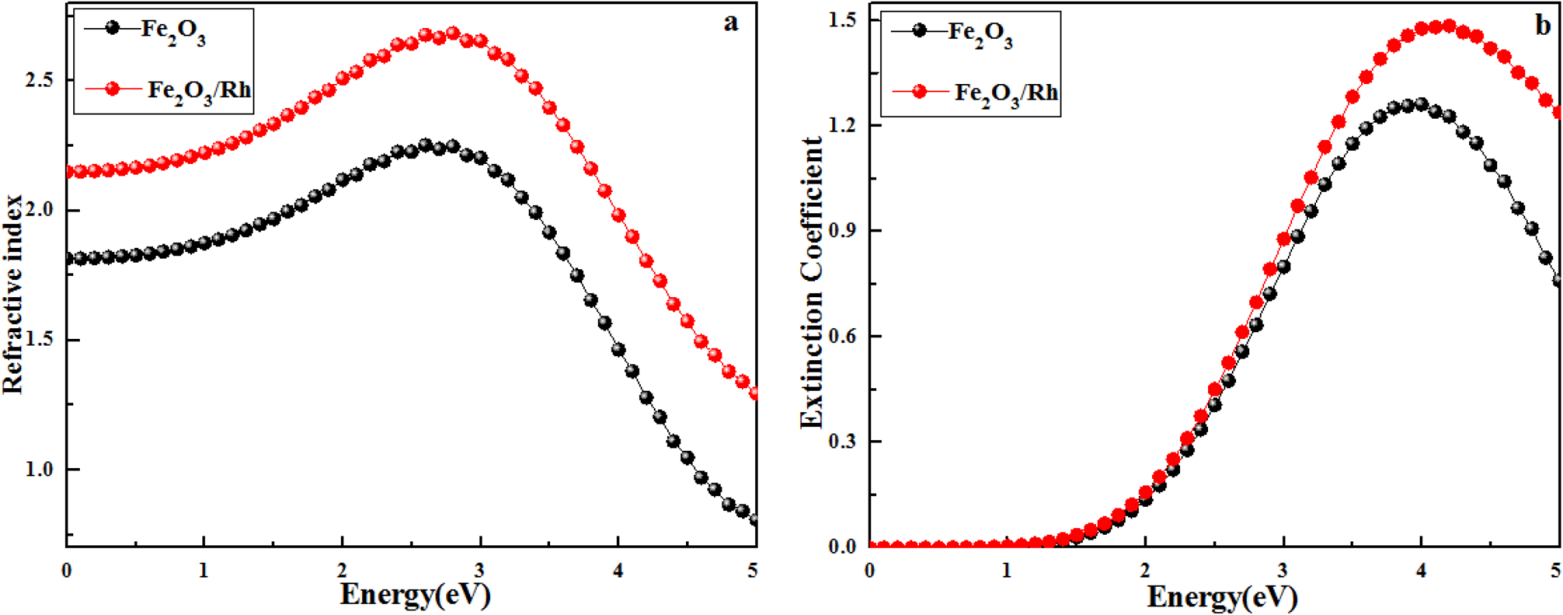
Refractive index (**a**) and extinction coefficient (**b**) of pure and doped hematite at different incident energies.

The refractive index depends on the physical properties of a material and the wavelength of propagating light. An increase in the refractive index of rhodium doped hematite in comparison with pure hematite (shown in Fig. 10a) indicates high density and polarizability of the material due to the rhodium 4d and 5s contributions which attenuate the propagating light^58^. As shown in Fig. 10b, an increase in the extinction coefficient indicates that the surface of rhodium doped hematite absorbs more light than the pristine hematite which allows for more permeation of light and hence improved optical properties of the material. The measured values of the refractive index and the extinction coefficient are given in Table 4.

**Table 4:** Refractive index (n) and extinction coefficient (k) in the visible region at incident E _photon_ = 1.65 eV ($\lambda$ $\approx$ 750 nm).

Material	n	Exp^59^	k	Exp^59^
Fe_2_O_3_	1.506	2.619	1.364	0.060
Fe_2_O_3_/Rh	1.740	–	1.393	–

**Figure 11:**
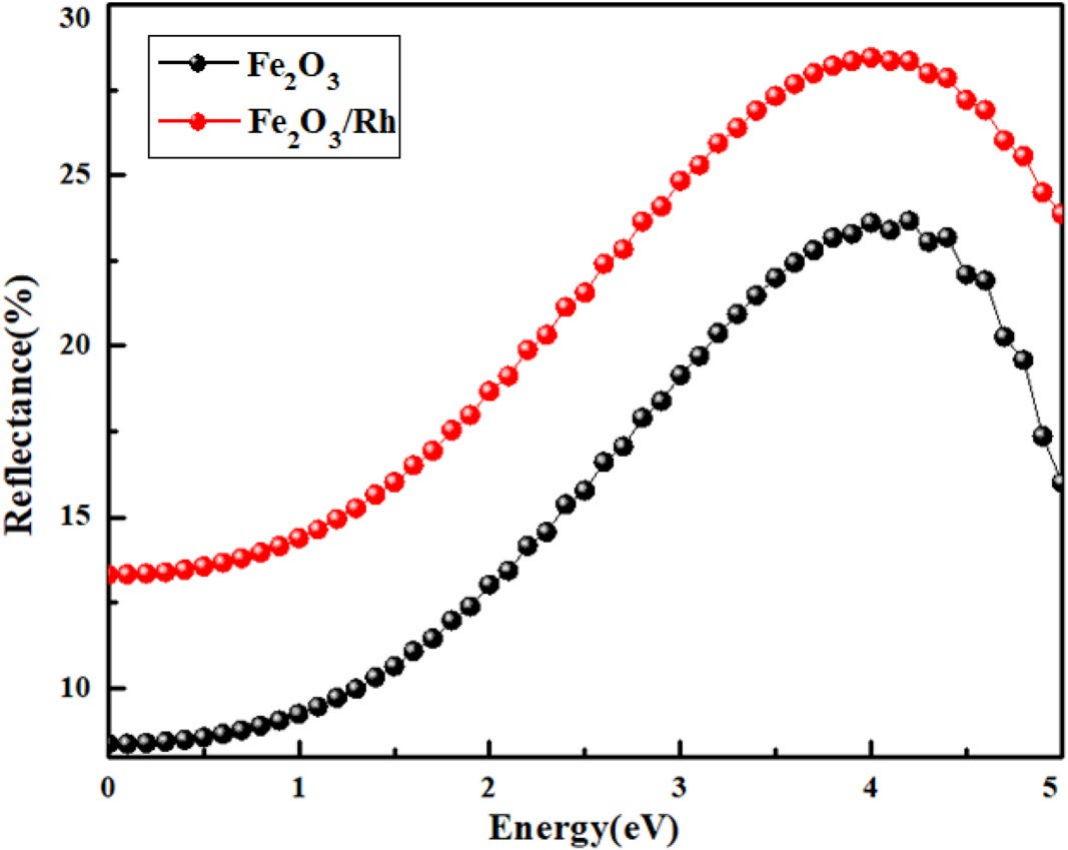
Comparative reflectance between pure and Rh doped hematite across the energy levels from visible to ultraviolet range of the electromagnetic spectrum.

The high reflectance of the rhodium doped sample (Fig. 11) is attributed to the redshift in the bandgap and the evolution of the metallic nature due to the substitution of rhodium in hematite. The maximum reflectance in the visible range (390 nm) was ~ 26.8% for rhodium doped hematite.

## Conclusions

To investigate the optoelectronic properties of rhodium doped hematite, DFT-based simulations were carried out. It was observed that a 3.3% rhodium incorporation in hematite enhanced the optical absorption in the visible to ultraviolet regions, and charge transport capability from infrared to the entire ultraviolet region of the solar spectrum. Rhodium doping in hematite lowered its bandgap to 1.61 eV making doped-hematite a promising candidate material for efficient photoelectrodes. The absorption capability of doped-hematite also improved substantially over a broad range of the solar spectrum (250–800 nm) as compared with pure hematite. The enhancement in optical absorption increased carrier concentration which subdues the electron–hole recombination, resulting in possibly high photocurrent and IPCE. On the other hand, the enhanced absorption in the lower visible region may allow hematite to utilize low energy photons for photoelectrochemical processes. The formation energy and free energy of the doped hematite was much lower than pure hematite indicating the thermodynamic stability of the material. The substitutional-doped hematite exhibited improved dielectric function, reflectance, extinction coefficient, and index of refraction demonstrating its technological potential in solar cells and optoelectronic applications.
